# Suitability of Paper-Based Substrates for Printed Electronics

**DOI:** 10.3390/ma15030957

**Published:** 2022-01-26

**Authors:** Elina Jansson, Johanna Lyytikäinen, Panu Tanninen, Kim Eiroma, Ville Leminen, Kirsi Immonen, Liisa Hakola

**Affiliations:** 1 VTT Technical Research Centre of Finland Ltd., Sensing Solutions, Kaitovayla 1, FI-90590 Oulu, Finland; 2 Laboratory of Production Engineering, Department of Mechanical Engineering, School of Energy Systems, LUT University, Yliopistonkatu 34, FI-53850 Lappeenranta, Finland; Johanna.Lyytikainen@lut.fi (J.L.); panu.tanninen@lut.fi (P.T.); Ville.Leminen@lut.fi (V.L.); 3 VTT Technical Research Centre of Finland Ltd., Sensing Solutions, Tietotie 3, FI-02150 Espoo, Finland; kim.eiroma@vtt.fi (K.E.); liisa.hakola@vtt.fi (L.H.); 4 VTT Technical Research Centre of Finland Ltd., Biomaterial Processing and Products, Visiokatu 4, FI-33720 Tampere, Finland; kirsi.immonen@vtt.fi

**Keywords:** paper-based electronics, printed electronics, re-pulpability

## Abstract

Flexible plastic substrates are widely used in printed electronics; however, they cause major climate impacts and pose sustainability challenges. In recent years, paper-based electronics has been studied to increase the recyclability and sustainability of printed electronics. The aim of this paper is to analyze the printability and performance of metal conductor layers on different paper-based substrates using both flexography and screen printing and to compare the achieved performance with that of plastic foils. In addition, the re-pulpability potential of the used paper-based substrates is evaluated. As compared to the common polyethylene terephthalate (PET) substrate, the layer conductivity on paper-based substrates was found to be improved with both the printing methods without having a large influence on the detail rendering. This means that a certain surface roughness and porosity is needed for the improved ink transfer and optimum ink behavior on the surface of the substrate. In the case of uncoated paper-based substrates, the conductivity and print quality decreased by preventing the formation of the proper and intimate ink-substrate contact during the ink transfer. Finally, the re-pulpability trials together with layer quality analysis detected very good, coated substrate candidates for paper-based printed electronics competing with or even outperforming the print quality on the reference PET foil.

## 1. Introduction

The demand for a variety of flexible electronics, such as wearable electronics, optoelectronics, flexible printed circuits, hybrid circuits, energy devices, and sensors, has been increasing rapidly in recent years. At the same time, roll-to-roll (R2R) and high-throughput manufacturing is becoming increasingly popular in the electronics industry, driving the search for novel flexible substrate materials suitable for printing processes [1]. However, currently, the situation in printed electronics is challenging from an environmental perspective due to commonly used plastic substrates, such as polyethylene terephthalate (PET) or polyethylene naphthalate (PEN), and metal inks, such as silver and copper. Indeed, the main sources of climate impacts and sustainability challenges in the printed electronics stem from fossil-based substrate materials and metals used [2,3]. Bio-based flexible substrates, such as papers and paperboards, with smaller environmental footprint and originating from renewable resources are clear opportunities to improve the situation. Many of these materials are often recyclable and biodegradable.

Paper-based substrates are of low cost, flexible, biodegradable, recyclable, and de-formable as well as often more thermally stable than commonly used flexible plastic foils [4,5,6,7]. However, their high roughness, absorbency, poor barrier properties, opaqueness, and sensitivity to moisture are considered to create challenges in printed electronics where ultra-smooth, dimensionally stable, and non-absorbing substrates are required for highly functional devices [5,6,7,8,9]. Fortunately, the roughness, porosity, and surface energy of paper-based substrates can be easily adjusted and controlled by means of coating, calendering, and adding fillers and additives [6,7]. Paper coatings, for example, increase surface smoothness and reduce the size of the pores. This way ink and moisture absorption and penetration into paper can be decreased through decreased fiber swelling and improved paper strength. At the same time, layer conductivity can be increased and printability as well as barrier properties improved [4,6,7,8]. On the other hand, too smooth and closed paper surface increase lateral ink spreading, significantly resulting in lower print resolution. This also decreases ink transfer, resulting in thinner ink layers and thus poorer conductivity and ink adhesion [8,10,11]. Despite the possibilities to easily affect and improve paper properties, higher electrical resistance is typically expected on papers than plastic foils [12]. Other flexible bio-based materials, such as biopolymer polylactic acid (PLA), silk fibroin, nanocellulose (NCF), and nanochitin, have also been developed for printed electronics applications where transparency and high smoothness of the flexible substrate are required [8,13,14,15,16].

Paper-based electronics is a widely studied area. Paper has been evaluated as a substrate for thermochromic and electrochromic displays, resistive memory devices, transistors, capacitors, disposable radio frequency identification (RFID) tags, batteries, photovoltaic cells, and sensors and actuators [4,5,6,12,17,18,19]. In recent years, the use of paper-based substrates in diagnostics, pharmaceutical, energy harvesting, and wearable applications has grown due to paper’s high breathability consequently from porosity, flexibility, and sustainability [6,20]. Examples of these devices include wearable pressure and humidity sensors, thermoelectric generators, as well as smart bandages [20]. Paper-based electronics have also been demonstrated with high-volume mass-manufacturing techniques. Hakola et al. [5] manufactured fully-printed electrochromic display elements onto paper substrate using R2R rotary screen printing with high yield and good performance. 

Printed conductive tracks are important building blocks for any electronic device, thus highlighting the good printability properties on paper substrates combined with good electronic performance. The most widely used printing methods with paper-based electronics include screen printing, gravure, flexography, and inkjet printing [7,8,10,20]. To avoid absorption or penetration of functional materials into the paper structure and to achieve high conductivity, particle-based conductive metal inks or pastes are typically used with paper-based substrates [6]. Although metal particulate inks are not considered sustainable materials as such, the development of deinking and recycling or reusing strategies should decrease their environmental footprint in the future [7]. Other opportunities arise from replacing harmful and critical metals with abundant metals, and by aiming at minimal use of metals in general.

Micro particle conductive inks contain micrometer-sized metal flakes mixed with binders, solvents, and additives [7,21,22]. After printing, the solvent is removed from the printed layer via evaporation at elevated temperatures of typically 100–150 °C. This way the conductive particles are packed closer together and the number of conductive paths within the printed layer increase. Micro particle inks give reasonable conductivity even after drying at room temperature, are less prone to penetrate into the paper pores, and are cheaper than nanoparticle inks [4,7]. Sheet resistance values of 40–50 mΩ/square have been reached with printed antennas on paper. However, due to the large flake size, low sheet resistance is achieved only with thick layers, which in turn increases the roughness and volume resistivity of the printed layers, and decreases the detail rendering [7,21,22].

Nanoparticle conductive inks, for their part, contain spherical metal nanoparticles that are encapsulated with a thin protective shell dispersed in solvent. [23,24,25]. After printing, a sintering step is added to remove the encapsulation layer and cause adjacent nanoparticles to coalescence. Typically, the sintering of the printed layers on flexible substrates is done with heat at elevated temperatures of 120–150 °C. By decreasing the amount of encapsulant material and increasing the particle size to 150–160 nm or using alternative encapsulation materials, the sintering temperature can be lowered, and high conductivity of 4.6 µΩ·cm can be achieved [25,26]. Other sintering methods have also been developed to enable the particle coalescence even at room temperature and to broaden the flexible substrate selection further. The most promising techniques are chemical sintering, where nanoparticles are destabilized with chemicals, and electrical sintering where an applied voltage causes current flow through the structure leading to local heating by dissipation [23,24,27]. On paper-based substrates, the reached volume resistivity values are 6.8 µΩ·cm and 2.7 µΩ·cm for chemical and electrical sintering, respectively.

Intelligent packaging is often mentioned as one of the most promising areas for paper-based electronics [9]. The goal of intelligent packaging technologies is to provide means for controlling packed product quality or informing about it, to provide more convenience to consumers, to market and brand the products, and to control counterfeiting and theft [28]. Printed electronics is often said to enable ‘electronics everywhere’ [29,30]. This causes a challenge to be able to collect and manage these devices from different waste streams, for example, among packaging waste that is a sector strongly relying on paper based materials. According to Furuta et al. [31] and Erdmann et al. [32], electronic components can influence the composition of solid and liquid residues in paper recycling process, and thereby affect disposal costs. The increasing amount of adhesives in the pulp coming from component assembly and electronic label attachment may result in an increase of agglomerated adhesives and fibers. Aliaga et al. evaluated how electronic components affect paper recycling [33]. Their observation was that the presence of printed electronic components did not increase the fiber rejects during paper recycling, and properties of the recycled paper were not significantly affected. Therefore, the recyclability of printed paper-based electronics seems to be mostly affected by the substrate itself.

In this paper, the printability of the silver conductor tracks on different paper-based substrates was analyzed using two commonly used printing methods in the printed electronics field, i.e., flexography and screen printing. Furthermore, the achieved print quality and conductor layer performance was compared to the printed layer properties achieved with the PET foil substrate that is commonly used as a flexible substrate in printed electronics. The re-pulpability of the selected paper-based substrates was also analyzed to determine the reusability potential of the substrates.

## 2. Materials and Methods

### 2.1. Substrate Selection and Analytics

Seven different commercially available paper-based substrates were selected for the printability trials: four printing papers (P1–P4), two coated paperboards (PB1, PB2), and one uncoated board (B1), as presented in Table 1. In order to evaluate the printability of the substrates in real life applications, their top side (TS) was printed meaning that the ultra-smooth side of P3, the smoother (outer) side of PB1, the uncoated (outer) side of cup stock PB2, and the smoother side of B1 were printed.

#### 2.1.1. Re-Pulping Experiments

The re-pulpability of the paper-based substrates was tested based on the Fibre Box Association’s (FBA) method [34] with small modifications. In total, 60 g of substrate was used in the experiments. After screening, the substrate material that did not pass through the sieve was collected as reject. The percentage of reject was calculated from the initial mass and the reject mass. The re-pulpability test is passed, and the material can be considered re-pulpable when the amount of reject is below 15%.

#### 2.1.2. Surface Roughness

The surface roughness of the printed side of the substrates was measured using a white-light interferometer Wyko NT3300 (Veeco, Plainview, NY, USA). Both arithmetic mean (R_a_) and root mean squared (R_q_) deviations of the surface profiles were recorded.

#### 2.1.3. Contact Angle

Substrate samples were conditioned at 23 °C and at 50% relative humidity before the contact angle measurements. Contact angle of water on the surface was measured using Theta optical tensiometer (Biolin Scientific, Gothenburg, Sweden). Deionized water was used in the measurements and the value was recorded after one second when the drop was applied on the surface of the substrate.

#### 2.1.4. Moisture Content

The moisture content of the substrates was measured according to SCAN P 4:63 (Paper and board – Determination of moisture content – Oven-drying method) standard. Substrate samples were conditioned at 23 °C and at 50% relative humidity before the measurement.

### 2.2. Printing Experiments

As already mentioned, conductor lines establish the backbone of the printed electronics and are practically needed in every application. Therefore, the printability and performance of the conductor lines are utmost important when evaluating the suitability of the more sustainable paper-based substrates for the printed electronics industry.

**Figure 1:**
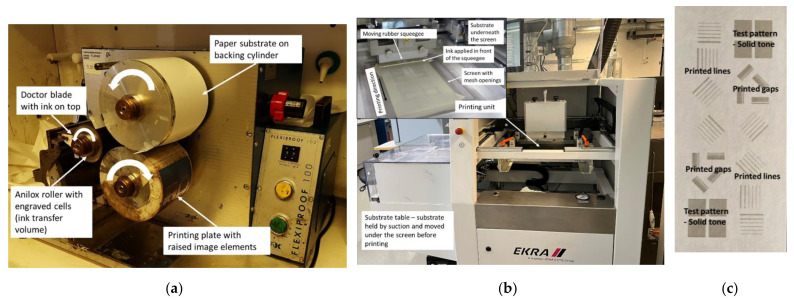
(**a**) Flexiproof 100 printer used for the flexographic printing trials and the principle of the flexography; (**b**) EKRA E2 screen printer and its operating principle, and (**c**) the printing layout.

The printing experiments were carried out using sheet-to-sheet (S2S) flexography and screen printing to deposit silver conductor lines onto the selected paper-based substrates. The layout contained simple conductor lines having different widths (50, 100, 150, 200, 300, 500, and 1000 µm) and orientations (horizontal, vertical, inclined), as well as densely packed conductor lines with gap widths of 50, 100, 150, 200, 300, 500, and 1000 µm. The narrowest 50 µm lines and gaps were excluded from the screen printing trials due to the resolution limitations of the utilized screen and the printing method itself. The utilized printing presses and layout are shown in Figure 1.

#### 2.2.1. Flexographic Printing Trials

The principle of the flexographic printing is shown in Figure 1a. The ink is initially applied onto a ceramic or chrome-plated anilox roller. The anilox roller has tiny cells with a certain volume engraved or etched evenly onto its surface. As the ink covers the surface of the anilox roller completely, a steel doctor blade removes excess ink so that the ink remains only inside the cells of the anilox roller. After this, the ink is transferred in a nip onto a flexible photopolymer printing plate that has the printed patterns as raised elements on its surface. Finally, the ink is transferred onto the substrate in a printing nip and dried. The cell volume of the anilox roller determine the amount of ink available during the process. The nip pressures and printing speed determine the final ink transfer volume onto the substrate and also the ink spreading, i.e., detail rendering.

Flexographic printings were done with Flexiproof 100 (RK PrintCoat Instruments, Litlington, UK) tabletop printer using PFI-722 water-based silver nanoparticle ink (Novacentrix, Austin, TX, USA). The viscosity of the ink is 300–600 mPa·s and silver content is 60 wt-%. The printing plate was Cyrel^®^ DPR 67 (DuPont, Wilmington, DE, USA) with a thickness of 1.7 mm and hardness of 69 ShA. The printing speed was 50 m/min and the printed layers were dried with hot air in a box oven at 130 °C for 30 min. Two different ceramic anilox rollers with the ink transfer volumes of 5 mL/m^2^ (200 cells/cm) and 7 mL/m^2^ (120 cells/cm) were used. The B1 board could not be printed with flexography due to its high stiffness and thickness that prevented its proper mounting around the substrate cylinder and its smooth passage through the printing process. In addition, a 125 µm thick PET plastic foil Melinex ST506 (DuPont Teijin Films, Chester, VA, USA) was printed as a reference substrate to compare the achieved printability results on paper-based substrates with the quality achieved on a substrate used commonly in printed electronics.

#### 2.2.2. Screen Printing Trials

In S2S screen printing (Figure 1b), the ink is applied in front of the rubber squeegee that squeezes ink through the screen openings onto the substrate. The screen is manufactured from metal or polymeric threads that form a mesh pattern with openings between the threads. The mesh count of the screen, thread parameters, and squeegee pressure determine the ink transfer amount and resolution. The non-image areas of the screen are covered with a stencil, thus leaving only image areas open and ready for the ink transfer. As the squeegee pushes the screen into contact with the substrate and moves along the screen, the ink gets transferred onto the substrate. After the printing, the printed layer is dried. The substrate is typically conveyed to the printing unit underneath the screen on a moving substrate table onto which the substrate is held in place by means of suction.

Screen printing trials were done with an E2 sheet-based screen printer (EKRA, Aachen, Germany) using LS-411AW micro particle silver paste (Asahi Chemical Laboratory Co.Ltd., Tokyo, Japan). The viscosity of the paste is 22 Pa·s, silver content approximately 70 wt-%, and solids content approximately 85 wt.%. A stainless steel screen (Koenen, Ottobrunn-Riemerling, Germany) with a mesh count of 325 lines/inch and a wire diameter of 24 µm was used to print the conductor layers at the speed of 40 mm/s. The utilized rubber squeegee had a hardness of 65 ShA and angle of 45°. The printed samples were dried with hot air in a box oven at 150 °C for 20 min.

#### 2.2.3. Rotary Screen Printing Trials

**Figure 2:**
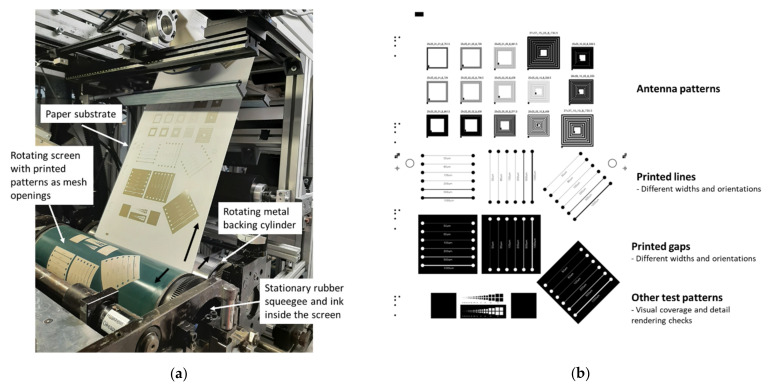
Roll-to-roll (R2R) rotary screen printing process with P1 paper using VTT’s R2R printing line and the operating principle of the rotary screen printing (**a**) and the layout for the R2R printing trials (**b**).

Figure 2a presents the operation principle of the roll-to-roll (R2R) rotary screen printing. The cylindrical metal screen having printed patterns as openings on its surface rotates to enable a continuous operation. Stationary rubber squeegee is located inside the rotating screen and the ink is also applied inside the screen. The ink gets transferred from the inside of the screen onto the substrate as the squeegee is pressed against the rotating screen and moving substrate. The metal backing cylinder acts as support for the substrate during the ink transfer. After the ink transfer, the substrate web with printed layers is conveyed to the drying section of the R2R printing line. 

Rotary screen printing trials in R2R environment were performed with VTT’s pilot production line (Figure 2a) onto P1 (100 g/m^2^) and P3 papers as well as onto PET plastic foil Melinex ST506 (DuPont Teijin Films, Chester, VA, USA). Microparticle silver paste LS-411AW (Asahi, Japan) was printed using cylindrical stainless steel screens (SPGPrints, Boxmeer, The Netherlands) with a mesh count of 305 lines/inch and theoretical wet ink layer thickness of 11 µm. The layout, shown in Figure 2b, consisted of printed lines and gaps with widths of 50, 80, 100, 200, 500, and 1000 µm as well as antenna patterns having line and gap widths of 100, 200, 500, and 1000 µm. The printing speed was 2 m/min and temperature of the hot-air ovens of ROKO was set to 140 °C. The total dwell time in the drying section was approximately 3 min. The print run length was approximately 30 m per substrate and the quality analysis was done for 5–10 m of samples.

### 2.3. Printability Analysis

The printability of the conductor lines on the paper-based substrates was evaluated considering their use in the printed electronics applications. Important printability parameters included the layer conductivity, i.e., the sheet resistance (SR) and volume resistivity (VR) of the printed conductor layers, minimum conductive conductor line width, minimum reproducible gap width, and ink spreading.

#### 2.3.1. Minimum Conductive Line Width

The minimum conductive line width was determined by measuring the resistance of the printed conductors having different widths and orientations with a common digital multimeter FLUKE 289 (Fluke, Everett, WA, USA). The narrowest line width that gave a measurable resistance value was evaluated to be the minimum reproducible line width that can be utilized in the printed electronics applications. The measurements were taken for every line orientation separately from which an average value of the minimum conductive line width in a test point was calculated.

#### 2.3.2. Detail Rendering

The minimum reproducible gap width and ink spreading was analyzed by means of the SmartScope ZIP 250 microscope (OGP, Rochester, NY, USA). The minimum gap width was determined by detecting the completely open gaps without any ink splashes in every pattern orientation separately and calculating the average minimum gap in a test point. The ink spread was determined by measuring the widths of 500 µm and 1000 µm wide lines in every orientation and calculating the difference between the measured and designed line widths. The measurements were taken in every line orientation separately and an average ink spreading in a test point was then calculated. In addition, the visual quality of the printed conductor layers was analyzed by means of NeoScope^TM^ JCM-5000 (JEOL, Tokyo, Japan) scanning electron microscope (SEM).

#### 2.3.3. Conductivity

The resistance of the printed 1000 µm wide lines with different orientations was measured with the common digital multimeter FLUKE 289 (Fluke, Everett, WA, USA). The layer thickness was measured with Dektak 150 surface profilometer (Veeco, Plainview, NY, USA) from samples where the ink did not penetrate into the surface irregularities of the substrate. Thus, the layer thickness was measured on PET and on P1 paper in the case of flexography and screen printing, respectively. In R2R screen printing, the layer thickness was measured from all the test points. The sheet resistance and volume resistivity were then calculated by means of the measured resistance (R), line width (w), line length (l), and layer thickness (h):

SR (Ω/sq.) = Rwl^−1^ (1)

VR (Ω·cm) = SR h(2)

## 3. Results

### 3.1. Paper-Based Substrate Quality

Table 2 presents the measured properties of the substrates. Roughness and contact angles were measured from the printed side of the substrates. The surface roughness of the substrate was found to influence the ink transfer so that rougher surfaces accept more ink but as the surface roughness increases too much, the ink–substrate contact area decreases, and the ink transfer gets reduced [35]. All the paper-based substrates used in this study have significantly higher roughness than the reference substrate PET, thus predicting differences in the ink transfer, printability, and performance of the conductor lines between the selected substrate types. The smoothest paper-based substrates are P3 and PB1 whereas the roughest substrates are P4 and B1.

The surface is considered hydrophobic, and ink wetting is lower when the contact angle of water is over 90°, as is for P3, P4, PB2, and B1. However, with paper-based substrates, the wetting is affected by porosity, localized hydrophobicity (internal sizing), and roughness of the substrate. In addition, the dynamic phenomena of the contact angle on paper-based substrates might depict the ink wetting phenomena more accurately than the static contact angle measured done here [36]. It should be noted that ink wetting and spreading are also affected by the forces present during the ink transfer in the printing nip [37].

The amount of reject is under <1% for P1, P2, PB1, PB2, and B1 substrates, thus indicating their excellent re-pulpability. However, the amount of reject is calculated from the material left on the sieve and this may be increased due to the coating, filler, and additives type, or if the tested material has, for example, larger particles or very long fibers and not entirely because of the presence of un-recyclable materials. For example, re-pulping of P3, P4, and PB2 substrates results in large amounts of rejects, thus indicating only the presence of larger particles/fibers or unsuitable materials for the utilized re-pulping method.

The moisture content of the paper-based substrate is the lowest for P2 and P3 papers and the highest for PB1 and PB2 paperboards. The lower the moisture content of the paper, the better its dimensional stability, thus improving the registration accuracy in multilayer printing. Typically, appropriate moisture content level of printing papers is 5–7%. Too high moisture content can create difficulties in paper runnability in the printing process and layer unevenness or poorer printability due to uneven ink absorption and ink transfer. In addition, too low moisture content, for its part, creates runnability issues by causing the paper to tear more easily during processing. According to the results, printability and dimensional stability issues might be present with PB1 and PB2 substrates that has higher moisture content than 7%. However, these materials are coated with a barrier layer that reduces the effect of the moisture content of the base paper to print quality, dimensional stability, and runnability. 

### 3.2. Printability in Flexography

**Figure 3:**
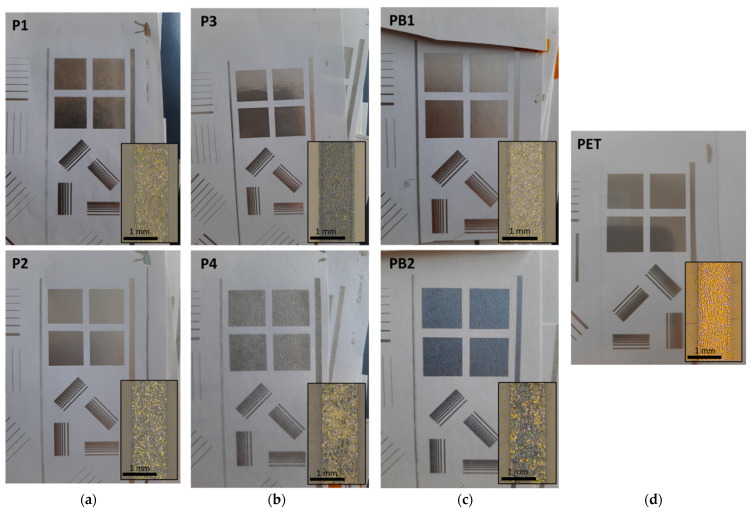
Photographs of the flexo-printed conductor layers on paper-based substrates using the nanoparticle silver ink and the ink transfer volume of 5 mL/m^2^. In addition, microscope images of the printed 1000 µm wide vertical lines with a scale bar of 1 mm are shown. The substrates are papers P1–P4 (**a**,**b**), paperboards PB1 and PB2 (**c**), and reference polyethylene terephthalate (PET) plastic foil (**d**).

All the selected paper-based substrates are properly printable with flexography and give good layer coverage with sharp printed edges and reproducible gap details, as shown in Figure 3. The layer quality on paper-based substrates is rather similar to the quality on the reference PET foil. On paper-based substrates, the edge raggedness or waviness is expectedly higher due to their more irregular surface. However, the layer coverage and line definition on coated paper-based substrates have equal quality as compared to the quality on the reference PET foil. Therefore, the higher layer roughness of the coated paper-based substrates does not adversely affect the print quality. However, the surface texture of the even rougher uncoated P4 and PB2 substrates shines through the printed layers, thus giving a more mottled appearance and seemingly decreasing the layer coverage. This results from the ink absorption into the pores, ink flow, or spreading along the surface irregularities, as well as uneven ink transfer, i.e., formation of poorer and inhomogeneous ink-substrate contact. The visual print quality remains unchanged as the ink transfer volume increases from 5 mL/m^2^ to 7 mL/m^2^. 

**Figure 4:**
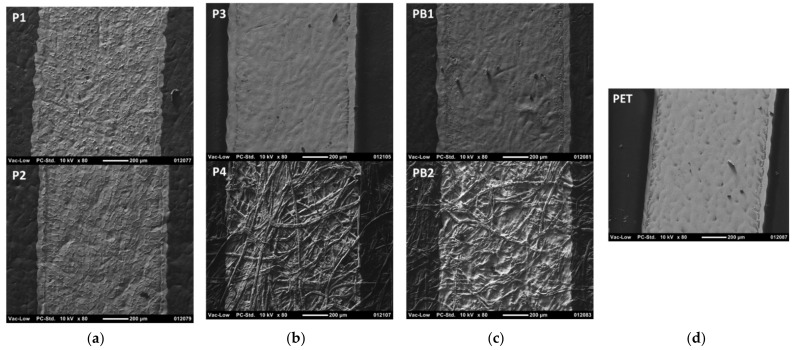
Scanning electron microscope (SEM) images of the flexo-printed conductor lines on paper-based substrates using the nanoparticle silver ink and the ink transfer volume of 7 mL/m^2^. The magnification is ×80 and the scale bar is 200 µm. The substrates are papers P1–P4 (**a**,**b**), paperboards PB1 and PB2 (**c**), and reference PET plastic foil (**d**).

**Figure 5:**
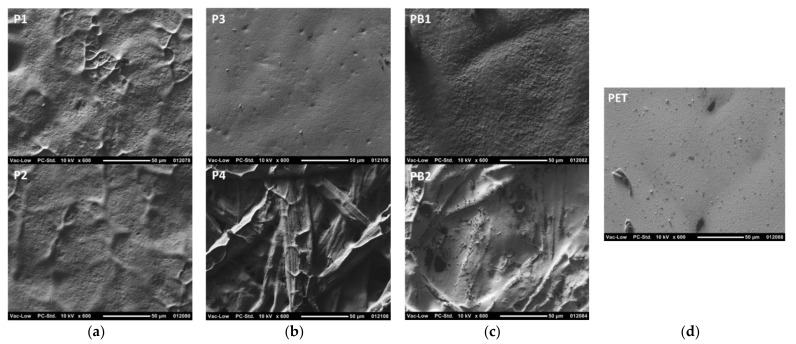
SEM images of the flexo-printed conductor lines on paper-based substrates using the nanoparticle silver ink and the ink transfer volume of 7 mL/m^2^. The magnification is ×600 and the scale bar is 50 µm. The substrates are papers P1–P4 (**a**,**b**), paperboards PB1 and PB2 (**c**), and reference PET plastic foil (**d**).

Figure 4 and Figure 5 show that the printed layers are properly transferred onto the substrate and remain on the surface of the substrates rather than penetrate into the pores of the paper-based substrates. Even with uncoated paper-based substrates, the nanoparticle ink does not penetrate inside the paper structure along the pores but tends to follow the irregularities of the substrate surface. Therefore, the printed layers are expected to have good conductivity. However, the surface texture of the paper-based substrates is clearly visible through the thin nanoparticle ink layer. The measured layer thicknesses on PET were 302 nm and 632 nm for the ink transfer volumes of 5 mL/m^2^ and 7 mL/m^2^, respectively. When the layer thickness is smaller than the roughness of the substrate, as with P1, P2, P4, and PB2 substrates, the ink layer follows the surface irregularities of the substrate. With the uncoated rougher substrates P4 and PB2, the ink transfer into the deep valleys of the surface profile seems to be partial and ink-free areas are seen in the bottom of the surface irregularities. However, with smoother P3 and PB1 substrates, the ink layer fills the smaller surface irregularities completely, which in turn leads to smoother conductor layers with more even surface quality. On the ultra-smooth and un-porous PET foil, for its part, some layer unevenness and pinholes appear, thus indicating that the optimum ink transfer and levelling in flexography seems to require some substrate surface roughness and different surface energy with the utilized water-based ink.

Considering the suitability of the paper-based substrates for the printed electronics applications, the achieved layer conductivity is considered to be the most important property. The flexo- printed layers on paper-based substrates have high and even conductivity, as shown in Table 3.

Even better conductivity is achieved on the coated P1, P2, P3, and PB1 substrates than on the reference PET substrate. Thus, coated paper-based substrates seem to accept more ink and have better layer uniformity with more particle-to-particle contacts despite their higher surface roughness and porosity, and a wide range of contact angle values, as compared to the reference PET substrate. On uncoated P4 and PB2 substrates, for their part, the sheet resistance value and its deviation are higher because of the poorer ink transfer into the roughness profile of the substrates and the ink flow or spreading into the deep surface irregularities of the substrate during the layer levelling, thus decreasing the number of conductive particle-to-particle contacts within the printed layers. As the amount of particle-to-particle contacts decrease, less particles are available for merging and coalescence into each other, thus decreasing the layer conductivity further. 

The sheet resistance values in flexography are rather high (>100 mΩ/square) resulting from the low layer thickness of 302–632 nm. However, the nanoparticle ink leads to a low volume resistivity value of 1.4–46 µΩ·cm, i.e., high conductivity. The values achieved on coated paper-based substrates and on PET are comparable to the volume resistivities achieved with previous studies presented in the Introduction chapter. The volume resistivity values on coated P1, P2, P3, and PB1 substrates are equal or even lower than on PET, thus indicating a high ink transfer and good particle-to-particle contact formation also on rougher paper-based substrates. On uncoated P4 and PB2 substrates, the volume resistivities are higher because of the ink sinking into the large surface irregularities, thus reducing the number of particle-to-particle contacts and particle coalescence possibilities. In addition, the increase in the ink transfer volume decreases both the sheet resistance and the volume resistivity values due to the thicker layers with more densely packed nanoparticles. Therefore, it can be concluded that coated paper-based is a good alternative to plastic foils without sacrificing layer conductivity and performance.

**Figure 6:**
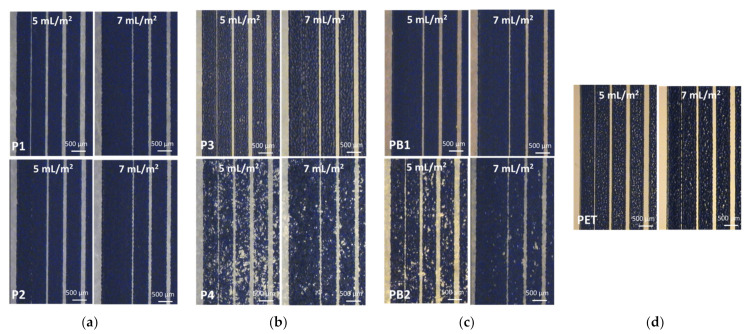
Microscope images of the flexo-printed gaps on paper-based substrates using the nanoparticle silver ink and two different ink transfer volumes. The scale bar is 500 µm and four narrowest vertical gaps, i.e., 50, 100, 150, and 200 µm, are presented. The substrates are papers P1–P4 (**a**,**b**), paperboards PB1 and PB2 (**c**), and reference PET plastic foil (**d**).

The reproducibility and detail rendering of the printed conductor layers play a major role in designing printed electronic circuits. Figure 6 shows the printed gap quality in the vertical direction using the two ink transfer volumes. As the ink transfer volume increases, the ink tends to spread more, thus increasing the minimum reproducible gap and decreasing the edge quality of the gaps. On uncoated paper-based substrates, the gap quality is equal to the other substrate since the surface irregularities prevent lateral ink from spreading. The gap quality seems to be poorer on coated P2 and PB1 substrates. As compared to the PET substrate, the minimum reproducible gap width on paper-based substrates is slightly increased, which indicates higher ink spreading on paper-based substrates. This higher spreading is most likely from the differences in surface energy between the paper-based substrates and PET as well as on higher ink transfer onto the paper-based substrates.

Table 4 presents the measured and calculated results for the different substrates and ink transfer volumes in flexography. The higher ink transfer volume of 7 mL/m^2^ increases the ink spread due to the increased amount of ink on the substrate. This also leads to the increased minimum gap width and decreased minimum conductive line. The ink spread and minimum gap widths are higher on paper-based substrates than on PET. However, the layer quality on P1 paper is not far from the quality on PET foil and the differences between the substrates are not that significant. There is no large difference in minimum conductive line between the paper-based substrates and the PET foil. However, coated P1 and uncoated PB2 substrates do not perform that well when printing these small details.

In conclusion, the selected paper-based substrates are excellent substrate candidates for printed electronics. Despite their higher roughness, the printed layer conductivity and coverage are improved, as compared to the printed layers on PET plastic foil. In addition, the detail rendering on paper-based substrates is at the same level as with PET foil. Therefore, some substrate roughness seems to be beneficial for proper ink transfer and formation of more partcile-to-partcile contacts within the printed layers. Uncoated paper-based substrates, for their part, suffer from ink penetration into surface irregularities, poorer ink transfer into deep surface irregularities, and ink flow along the surface irregularities, thus decreasing layer conductivity and visual quality. However, the deep surface irregularities prevent lateral ink spread, thus giving good detail rendering.

### 3.3. Printability in Screen Printing

**Figure 7:**
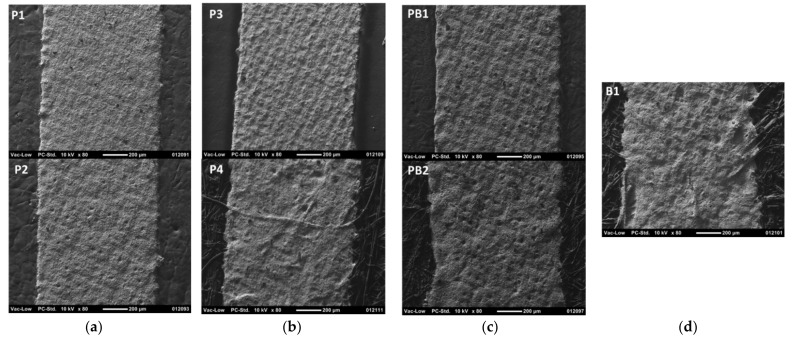
SEM images of the screen-printed conductor lines on paper-based substrates using the micro-particle silver paste. The magnification is ×80 and the scale bar is 200 µm. The substrates are papers P1–P4 (**a**,**b**), paperboards PB1 and PB2 (**c**), and board B1 (**d**).

As in the case of flexography with nanoparticle ink, micro particle silver paste is properly printable with screen printing onto different paper-based substrates (Figure 7). The higher solid content and viscosity of the conductor ink in screen printing increase the layer thickness to 12.6 µm, as compared to 302–632 nm in flexography. With coated substrates P1, P2, P3, and PB1, the surface irregularities of the substrate are completely filled with ink and the layer coverage is good, but the mesh pattern of the screen is copied onto the surface of the printed layers. The printed line edges become more ragged and wavier, and some surface texture and fibers of the substrate are seen through the printed layer with uncoated substrates P4, PB2, and B1. This results from the ink flow along the surface irregularities and fibers of the substrates as well as from the poorer ink transfer, i.e., the poorer contact formation between the ink/screen and the substrate.

**Figure 8:**
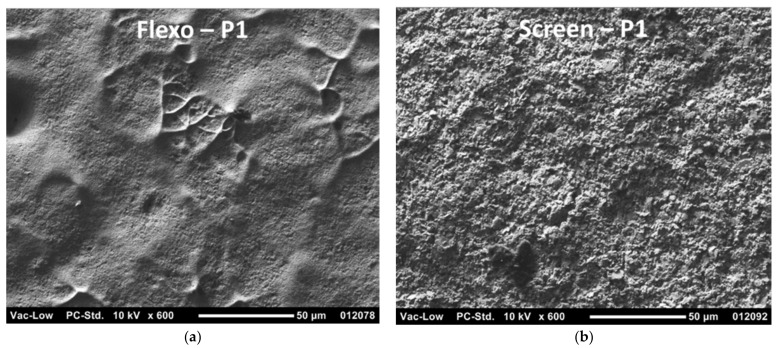
SEM images of the printed conductor layers on P1 substrate. The magnification is ×600 and the scale bar is 50 µm. The flexo-printed layer with nanoparticle ink (**a**) and screen-printed layer with microparticle ink (**b**) are shown.

The difference between the nanoparticle ink used in flexography and the microparticle ink used in screen printing is clearly visible in Figure 8. Tiny nanoparticles have merged and sintered together during the oven drying, thus forming a uniform layer and without seeing separate silver particles anymore. On the other hand, with the microparticle ink, separate silver flakes are clearly seen since no sintering takes place during the layer drying. Therefore, the printed layer has a more irregular and uneven surface, and its volume resistivity is expected to be higher.

Table 5 presents the properties of the screen-printed conductor layers on different paper-based substrates. The screen-printed layers have very low sheet resistance values (<40 mΩ/square) due to high layer thickness of 12.6 µm. However, as expected, the volume resistivity values are higher than with flexography resulting from the larger particle size in the ink and the un-sintered layer structure, thus forming less contacts between conductive particles within the printed layers. The substrate has no large effect on conductivity due to the thick layer covering and filling the surface irregularities and forming a continuous ink layer. On uncoated PB1 and B1 substrates, the ink spread and minimum gap widths are larger because of the ink flow along the surface irregularities. However, on uncoated P4, the ink spread is very low, despite the larger minimum gap value. This indicates that the printing of the densely spaced lines is more difficult and more prone to ink splashes and unevenness as well as excessive flow on the surface of the substrate than the printing of individual lines far apart from each other. The minimum conductive line is not affected by the substrate to a great extent. Only with B1, the minimum conductive line is 200 µm whereas with other substrates, the minimum line width is less than 150 µm.

Screen printing can reproduce thick conductor layers with good coverage and low sheet resistance onto different paper-based substrates. The thick printed layers can easily fill the surface irregularities of the substrate, thus giving low sheet resistance, despite the substrate roughness. However, on uncoated substrates, the ink tends to spread more along the surface irregularities of the substrate, thus making the detail rendering poorer and creating more ragged and wavier printed edges. The large particle size of the micro particle ink and the un-sintered layer structure after drying increases the achievable volume resistivity by decreasing the amount of conductive paths within the printed layer.

### 3.4. R2R Printability in Screen Printing

**Figure 9:**
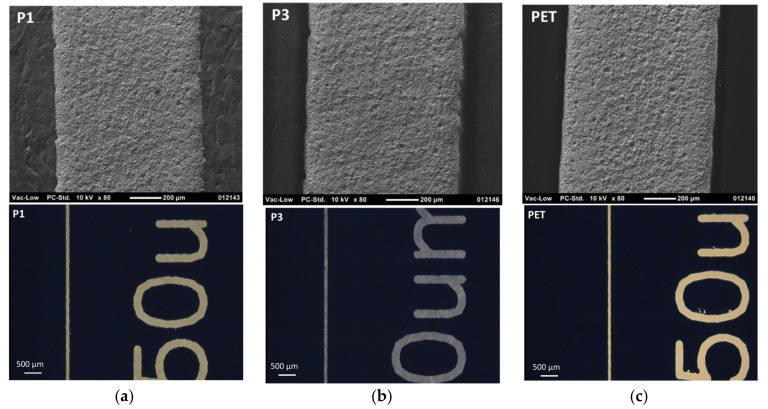
SEM images of the R2R screen-printed conductor lines on the selected substrates using micro-particle silver paste. The magnification is ×80 and the scale bar is 200 µm. The substrates are P1 (**a**), P3 (**b**), and PET reference foil (**c**).

Figure 9 shows good printability and detail rendering in R2R rotary screen printing on P1, P3, and PET substrates. The mesh pattern of the screen is visible on the surface, as with the S2S screen printing, resulting from the high ink viscosity that slows down the ink layer levelling. The layer conductivity is higher on paper-based substrates than on PET, although there are no large differences in layer thickness, as shown in Table 6. Therefore, the ink particles can align themselves into better contact with each other on rougher and more porous surfaces than on the extremely smooth and non-absorbent PET. The substrate has no effect on the detail rendering in the R2R printing since no uncoated substrates were used. Thus, the detail rendering is very good, and the ink layers do not spread on the substrates after the ink transfer in the R2R process.

As compared to the S2S screen-printed layer quality, the sheet resistance values in rotary screen printing are slightly higher because of the thinner ink layer, lower drying temperature, and shorter drying time. However, no difference in volume resistivity is seen, meaning that the screen-printed layers have similar conductivity but the differences in the screen properties and printing mechanisms create changes in the ink transfer amount. The detail rendering in rotary screen printing is better than in S2S screen printing. This can be explained by the smaller ink transfer and thinner layers in the rotary screen printing.

## 4. Discussion

The tested commercial paper-based substrates found to be suitable substrate candidates for printed electronics applications using both flexography and screen printing. Despite their higher roughness and porosity, as compared to PET foil and a wide range of measured contact angle levels, print quality and layer performance was equal or even better than on the reference PET substrate. Therefore, some layer roughness and porosity was found to be beneficial for ink transfer and formation of highly conductive layers. Previous studies stated that printed layer conductivities on papers have not reached the conductivity levels on plastic foils. However, in this paper, the quality and performance of the printed conductor layers on coated paper-based substrates was better or at the same level than on the PET foil, resulting from better ink transfer onto the rougher but rather even surface. In addition, the utilized commercial papers have modern coatings optimized for the printing of small details without ink penetration, and some grades are said to be even tailored for printed electronics by manufacturers, such as P3.

In flexography, the layer conductivity and coverage were higher on coated paper-based substrates than on the PET foil. This means that some layer roughness was needed to increase the ink transfer rate. The detail rendering properties were slightly poorer on the paper-based substrates than on the PET foil but the differences were rather small. The deep surface irregularities of uncoated substrates prevented lateral ink spread, thus improving the detail rendering properties—similar between the coated paper-based substrates and the PET foil. However, with rougher uncoated papers, the ink transfer got poorer, and the ink layer sunk into the irregularities of the paper surface, thus decreasing the conductivity. The contact angle of water on the paper-based substrates had no effect on the layer quality due to the high nip pressure forcing the ink to transfer and spread on the surface of the substrates.

The ink transfer volume in flexography had a large effect on the printed conductor layers. As the ink transfer volume increased, more ink was transferred onto the substrates, resulting in better conductivity but higher ink spreading and poorer detail rendering.

In screen printing, the effect of the substrate on the layer quality was small. The thick micro particle ink layers filled the surface irregularities, thus forming highly conductive layers, despite the substrate properties. However, the detail rendering was poorer with uncoated paper-based substrates due to the ink flow or spreading along the surface fibers and irregularities of the substrate. R2R printing trials also confirmed that better conductivity was achieved on coated paper-based substrates having higher roughness than on the ultra-smooth PET foil. Furthermore, no effect on the detail rendering could be detected.

The nanoparticle ink used in flexography formed uniform layers where individual particles had merged/sintered together, thus resulting in low volume resistivity. However, the resulting thin layer increased the sheet resistance. With microparticle ink used in screen printing trials, the individual silver flakes/particles remained separated, thus increasing volume resistivity. The high layer thickness, for its part, ensured the formation of low sheet resistance.

According to the re-pulpability and printability trials, the most promising recyclable substrates to be used in printed electronics applications were P1, P2, and PB1. These paper-based substrates were coated with an average surface roughness, R_a_, between 0.72 µm and 1.14 µm. The lower moisture content with P1 and P2 papers indicate their better dimensional stability during processing as well as more even print quality, as compared to the PB1 paperboard. Further considering the performance and quality of the printed layers, the most promising paper-based substrate is P1 when searching for an easily re-pulpable or recyclable alternative to PET plastic foil.

The results shown in this paper give encouraging data for the possibility to replace plastic foil substrates in printed electronics applications with coated, rough, porous, and recyclable paper-based substrates without sacrificing layer performance. The achieved results can be exploited when replacing plastic foils in applications where highly conductive or thick layers are needed, such as printed antennas, sensors, and displays. The flexible nature of paper-based substrates together with their good breathability (porosity) also open possibilities in wearable electronic applications. 

## Figures and Tables

**Table 1:** Selected substrate grades for the printability studies together with their grammage. TS = top side.

Code	Type	Grade	Grammage (g/m^2^)
P1	Coated paper	Stora Enso NovaPress Silk	80
P2	Coated paper	UPM Finesse Premium Silk	150
P3	Coated paper, ultra-smooth TS for printed electronics	Arjo Wiggins PowerCoat HD	95
P4	Uncoated paper, high durability	Paptic (Experimental Grade)	140
PB1	2-sided coated paperboard, TS (outer side of package)	Kotkamills Aegle^®^ White	180
PB2	1-sided coated paperboard for cup stock, TS uncoated	Kotkamills Isla^®^ Duo	195
B1	Uncoated board, TS smoother	Pankaboard PankaSilk	360

**Table 2:** Roughness, percentage of rejects, moisture content, and contact angle of water. Roughness and contact angle were measured from the printed side of the substrates.

Code	Roughness (µm)		Percentage of Rejects (%)	Moisture Content (%)	Contact Angle (°)
	R_a_	R_q_			
P1	0.97 ± 0.15	1.25 ± 0.25	0.0	6.6	65
P2	1.14 ± 0.18	1.49 ± 0.26	0.1	4.6	83
P3	0.20 ± 0.04	0.28 ± 0.06	27.3	5.5	98
P4	5.19 ± 0.91	6.77 ± 1.16	33.7	6.8	112
PB1	0.72 ± 0.07	0.93 ± 0.09	0.2	7.9	68
PB2	3.87 ± 0.46	5.10 ± 0.56	13.9	7.8	114
B1	5.34 ± 1.23	6.82 ± 1.60	0.6	7.0	119
PET (REF)	0.007 ± 0.002	0.013 ± 0.005	-	-	-

**Table 3:** Calculated average and standard deviation values for sheet resistance (SR) and volume resistivity (VR) of flexo-printed conductor layers on different substrates. The layer thickness used in the VR measurement was considered to be equal on every substrate since the high surface roughness of the paper-based substrates prevented reliable measurement of layer thickness.

Code	Sheet Resistance (mΩ/square)		Volume Resistivity (Ω·cm)	
	5 mL/m^2^	7 mL/m^2^	5 mL/m^2^	7 mL/m^2^
P1	326 ± 35	177 ± 19	9.8 × 10^−6^	2.2 × 10^−6^
P2	202 ± 22	138 ± 7	6.1 × 10^−6^	1.4 × 10^−6^
P3	253 ± 26	169 ± 16	7.6 × 10^−6^	1.6 × 10^−6^
P4	644 ± 111	318 ± 38	1.9 × 10^−5^	7.0 × 10^−6^
PB1	198 ± 27	177 ± 17	6.0 × 10^−6^	1.7 × 10^−6^
PB2	1524 ± 345	1199 ± 325	4.6 × 10^−5^	2.2 × 10^−5^
PET (REF)	356 ± 33	260 ± 23	1.1 × 10^−5^	2.1 × 10^−6^

**Table 4:** Measured ink spreading, minimum reproducible designed gap width, and minimum conductive designed line width of the flexo-printed conductor layers on different substrates.

Code	Spreading (µm)		Minimum Gap Width (µm)		Minimum Conductive Line Width (µm)	
	5 mL/m^2^	7 mL/m^2^	5 mL/m^2^	7 mL/m^2^	5 mL/m^2^	7 mL/m^2^
P1	4	51	75	100	103	106
P2	27	70	100	108	83	79
P3	13	41	100	108	83	79
P4	27	84	100	133	142	92
PB1	60	84	100	100	79	50
PB2	15	70	100	150	79	50
PET (REF)	13	34	67	100	78	67

**Table 5:** Calculated sheet resistance and volume resistivity as well as measured ink spreading, minimum reproducible designed gap width, and minimum conductive designed line width of the screen-printed conductor layers on different substrates.

Substrate	Sheet Resistance (mΩ/square)	Volume Resistivity (Ω·cm)	Spreading (µm)	Minimum Gap Width (µm)	Minimum Conductive Line (µm)
P1	30.8 ± 1.7	3.9 × 10^−5^	32	150	106
P2	35.4 ± 1.6	4.5 × 10^−5^	24	161	122
P3	33.1 ± 2.0	4.2 × 10^−5^	33	144	128
P4	31.0 ± 1.0	3.9 × 10^−5^	49	211	133
PB1	36.4 ± 1.0	4.6 × 10^−5^	57	128	106
PB2	38.8 ± 2.0	4.9 × 10^−5^	102	300	100
B1	32.7 ± 3.3	4.1 × 10^−5^	124	611	200

**Table 6:** Calculated sheet resistance and volume resistivity as well as measured layer thickness, ink spreading, minimum reproducible designed gap width, and minimum conductive designed line width of the rotary screen-printed conductor layers on different substrates.

Substrate	Sheet Resistance (mΩ/square)	Volume Resistivity (Ω·cm)	Thickness (µm)	Spreading (µm)	Minimum Gap Width (µm)	Minimum Conductive Line (µm)
P1	45.3 ± 1.3	4.1 × 10^−5^	9.1 ± 0.8	−27	50	87
P3	39.4 ± 0.6	3.4 × 10^−5^	8.5 ± 0.9	−26	50	87
PET	52.3 ± 2.5	4.7 × 10^−5^	8.9 ± 0.3	−19	50	98

## Data Availability

The authors confirm that the data supporting the findings of this study are available within the article. The raw data that support the findings of this study are available from the corresponding author, E.J., upon reasonable request.
